# Editorial: Dietary Antioxidants and Metabolic Diseases

**DOI:** 10.3389/fnut.2021.617859

**Published:** 2021-02-19

**Authors:** Daniele Vergara, Egeria Scoditti, Azlina Abdul Aziz, Anna M. Giudetti

**Affiliations:** ^1^Department of Biological and Environmental Sciences and Technologies, University of Salento, Lecce, Italy; ^2^National Research Council (CNR) Institute of Clinical Physiology, Lecce, Italy; ^3^Department of Molecular Medicine, Faculty of Medicine, University of Malaya, Kuala Lumpur, Malaysia

**Keywords:** dietary nutrients, cardiometabolic diseases, inflammation, oxidative stress, type 2 diabetes

The global and alarming increase in the incidence of metabolic disorders, including obesity, type 2 diabetes, and cardiovascular disease, and the related burden of morbidity and mortality highlight an urgent need for improved preventive and therapeutic strategies. Diet has a profound impact on human health as diet-associated metabolic disorders, such as obesity and type 2 diabetes, may be important risk factors for the pathogenesis of other diseases including metabolic syndrome and cancer. Thus, dietary habit represents a cornerstone in the prevention and treatment of many preventable chronic diseases.

Changes in food consumption patterns observed in the last decades to higher consumption of ultra-processed foods, with elevation in simple sugar and saturated fat contents and lower consumption of vegetables and fruits have been directly correlated with the metabolic syndrome prevalence (1, 2). Although various drugs are available for the treatment of metabolic complications, alternative treatments and approaches are constantly being sought, including a healthy balanced diet. Bioactive compounds from the diet possess many beneficial effects such as antioxidant activities which can be protective against chronic diseases.

Given the enormity of these clinical problems, this special issue aims to provide an update of the current knowledge of dietary bioactive compounds and their roles in human metabolic disease development. Specifically, this special issue includes a series of reviews and original articles, including animal and clinical studies. Such knowledge could pave the way for the development of dietary interventions for optimal health and prevention of metabolic diseases.

Esteve reviewed the role of cruciferous and their sulfurous components, called glucosinolates, in preventing metabolic diseases. Glucosinolates are inactive biologically in the organism but are hydrolyzed by the enzyme myrosinase released as a result of chewing, leading to the formation of active derivatives such as isothiocyanates and indoles. The authors collected a series of data on the effects of glucosinolate derivatives in modulating different pathways related to metabolic syndrome and cancer.

Alternative approaches are constantly sought for the treatment of metabolic diseases, including a healthy and balanced diet and the intake of specific nutrients with nutraceutical properties. In this regard, several experimental and clinical studies have been conducted to demonstrate the protective effects of dietary antioxidants against chronic diseases. However, many of these studies have produced conflicting data and there are currently no unambiguous data on the protective effects of these molecules. Therefore, the real impact of these bioactives on human subjects and their mechanism of action remain to be elucidated.

Spirulina and turmeric, in the form of nutraceuticals, are believed to exert not only an antioxidant effect but also modulate metabolic pathways that play a key role in the onset of cardiometabolic diseases. Gómez-Téllez et al. investigated the effects of treatment with spirulina maxima/turmeric longa mixture (266/156.6 mg) for 12 weeks in patients with abdominal obesity. The study reported an increased serum antioxidant status in patients supplemented with spirulina vs. placebo.

Polyphenols are bioactive compounds with anti-inflammatory, anti-obesity, and anti-aging properties (3). It is not completely clear whether other factors also contributed to these beneficial effects. Llaha and Zamora-Ros reviewed 15 randomized clinical trials to investigate the combined effects of polyphenol supplementation with caloric restriction and physical activity on body weight and fat, body mass index and waist circumference in overweight or obese adults. In these cohorts, isoflavone supplementation may increase fat loss during exercise among postmenopausal women of non-Asian ethnicity, thus supporting a possible favorable effect after polyphenol supplementation. However, these data should be interpreted cautiously because they do not support the effectiveness of caloric restriction and physical activity in combination with polyphenol supplementation on weight and fat loss. The authors concluded that isoflavone and soy products, combined with lifestyle changes, especially exercise, provided additional beneficial anti-obesity effects in postmenopausal women of non-Asian ethnicity. However, the potential role of polyphenols alone or in association with conventional therapies (caloric restriction and physical activity) mostly remains uncertain.

Resveratrol is a polyphenol with a plethora of biological effects. The molecule has a low solubility in water that favors the propensity for intestinal rather than gastric absorption. To ameliorate the solubility and the availability of resveratrol, Iannitti et al. developed and tested in a rabbit model, a solid dispersion of resveratrol supported by magnesium dihydroxide formulation (Resv@MDH). This formulation displays increased water solubility and better bioavailability in comparison to pure resveratrol. The pharmacokinetics profile was also studied in the whole blood samples of six healthy human subjects supporting a main gastric route for resveratrol absorption from Resv@MDH.

Dietary macronutrients such as carbohydrates and proteins, other than lipids, can influence the risk of metabolic syndrome, diabetes and fatty liver. Thus, carbohydrates, in particular in the form of simple sugar, like the sucrose that we generally use as a sweetener, can affect body composition and induce fatty liver. In light of this, Jang et al. conducted a study on animals and humans to investigate how palatinose-based alternative sweeteners, with increased sweetness, can contribute to blood glucose elevation. Three different preparations of palatinose-based sweeteners were tested: Palatinose-L, obtained from the reaction between sugar and the primary enzyme Protaminobacter rubrum CBS 574.77, Palatinose-IS derived from Palatinose-L treated with invertase, and Palatinose-FOS that was prepared from Palatinose-L treated with fructosyltransferase. *In vivo*, blood glucose levels at 60 min after the oral intake of Palatinose-based alternative sweeteners were significantly lower than those observed after the oral intake of glucose. Moreover, Palatinose-based alternative sweeteners were also characterized by a lower glycaemic index compared to glucose. These positive findings led to the conclusion that, due to the slow hydrolysis rate of palatinose-based sweeteners, the postprandial blood sugar can remain low thus helping to maintain a healthy status.

Dietary fatty acids, particularly as saturated fatty acids, represent prominent risk factors for metabolic disorders. In several observational studies, a controversial functional association has been reported between serum monounsaturated fatty acids (MUFAs) and cardiovascular events. In the study by Mazidi et al., the casual association of genetically determined serum MUFAs with coronary heart disease (CHD), myocardial infarction (MI), cardioembolic stroke (CS) and ischemic stroke (IS), was evaluated by Mendelian randomization analysis. No association was observed between serum MUFAs and the risk of CHD, MI, CS, and IS.

Collectively, the articles comprising this Research Topic provide novel insight into the impact of micronutrients, macronutrients, and functional foods on fundamental biological pathways, adding further knowledge and research opportunity for the development of new therapeutic options for metabolic disease prevention and treatment.

## Author Contributions

All authors listed have made a substantial, direct and intellectual contribution to the work, and approved it for publication.

## Conflict of Interest

The authors declare that the research was conducted in the absence of any commercial or financial relationships that could be construed as a potential conflict of interest.
